# Symptom specific deep brain stimulation for depression

**DOI:** 10.1016/j.neurot.2025.e00637

**Published:** 2025-07-07

**Authors:** Martijn Figee, Patricio Riva Posse, Helen Mayberg

**Affiliations:** a Nash Family Center for Advanced Circuit Therapeutics, Icahn School of Medicine at Mount Sinai, USA; b Department of Psychiatry and Behavioral Sciences, Emory University School of Medicine, USA

Deep brain stimulation (DBS) is an effective, safe, and well-tolerated therapeutic option for patients with severe treatment resistant depression (TRD) although not yet approved by any regulatory body. Despite halted trials for the various DBS targets under study, long-term outcomes in open label continuation studies report sustained long-term benefits despite the chronicity and the degree of treatment resistance in these cohorts [[1], [2], [3], [4], [5]]. Similarly, in a recent meta-analysis, half of a total of 275 patients with severe refractory depression could be classified as long-term responders after DBS [6]. Based on these reports, the FDA granted breakthrough device designation to re-investigate the long-term efficacy of subcallosal cingulate DBS in the United States in 2022 with a recently launched multicenter trial (TRANSCEND NCT06423430), and a pivotal trial of medial forebrain bundle DBS is seeking a Conformité Européenne (CE) mark in Europe (FORESEE III, NCT04021823).

These and other ongoing experimental trials have incorporated some important lessons learned from the past that will likely help optimize long-term efficacy of DBS for TRD. First, different from conventional antidepressant treatments, DBS modulates specific brain circuits requiring millimeter anatomical precision. Therefore, new trials have incorporated reproducible methods for surgical targeting of the implanted electrode based on connectomic tractography imaging. Second, new trials focus on the unique capacity of DBS to induce a slow but durable improvement in symptoms, adopting trial designs that allow for sufficient time to optimize stimulation parameters that putatively induce neuroplastic changes in brain circuits affected by chronic illness. For example, TRANSCEND focuses on symptom improvement measured over one year of active vs. sham stimulation. Complementary studies are evaluating biomarkers that might explain the variable trajectory of these responses and mechanisms mediating the observed durable effects. Finally, it is possible that different DBS targets may preferentially impact specific depression symptoms acutely with other symptoms changing more slowly over time. It is also becoming clear, as with DBS for Parkinson's disease or obsessive compulsive disorder, that not all symptoms may respond to all DBS targets or may require adjunct treatments with medication, psychotherapy or other rehabilitation strategies. However, symptom specific effects of different DBS strategies have been hampered by expectation of classical response metrics measured using standard symptom scales.

In this issue of Neurotherapeutics, Runia and colleagues present an analysis of symptom response patterns in DBS for TRD using a factor analysis of outcomes on the Hamilton Depression (HDRS) and Montgomery-Asberg depression (MADRS) rating scales, two of the most applied clinical depression scales [7]. The authors retrospectively examined long-term symptom recovery trajectories in 34 TRD patients receiving DBS in the ventral anterior limb of the internal capsule (vALIC) or superolateral branch of the medial forebrain bundle (slMFB). Between 56 ​% and 82 ​% of TRD patients responded to DBS at any follow-up time. However, these high response rates were not the focus of this study. Instead, the authors focused on symptom dimensions derived from previously published factor analyses of TRD patients responding to electroconvulsive therapy or ketamine: 1) affective/anhedonia, 2) somatic/anxiety, 3) insomnia, and 4) hopelessness. Between presurgical baseline and 2–15 years of vALIC or slMFB DBS, patients first experienced improvements in specific core depressive symptoms, such as mood, anhedonia and hopelessness, before slower changes in anxiety, somatic symptoms and insomnia.

The vALIC and slMFB targets used for DBS in this study are part of an interconnected frontostriatal circuit involved in motivation and reward processing, which may explain why DBS at these targets primarily elicited acute positive mood and improvement in anhedonia. The most widely used antidepressant DBS target, the subcallosal cingulate area (SCC), shares with vALIC and slMFB targets certain frontal and ventral striatal connections. This may explain why SCC DBS, also primarily changes mood before anxiety or other more secondary depressive symptoms such as insomnia or somatic symptoms [1,[8], [9], [10]]. However, the SCC DBS target also selectively connects to cingulate cortex connections into default and motor action mode networks. These unique SCC connections may explain the acute changes in negative mood and psychomotor slowness observed after SCC DBS [1], while not typically reported with the vALIC or slMFB targets. However, comparable to SCC DBS, with accelerated TMS targeted at SCC connectivity via the dorsolateral prefrontal cortex, primary changes in psychomotor slowness or lassitude have been reported [11]. Another DBS target recently examined for depression is the bed nucleus stria terminalis (BNST), which relative to the other targets may connect more directly to amygdala and dorsal anterior cingulate and was reported to benefit patients with anxious depression [12]. Symptom specific antidepressant targets for mood versus anxiety are also suggested for TMS [13]. Even conventional antidepressant treatments show greater improvements in core depressive symptoms than secondary symptoms. For example, residual anxiety and insomnia are often observed after remission from core depressive symptoms with medication or psychotherapy, and these residual secondary symptoms are strong predictors of depressive recurrence even in patients in remission [14,15]. Therefore, patients with recurrent refractory depressive illness no longer responding to conventional interventions may require treatment with DBS allowing for a more direct and durable modulation of circuits underlying their core depressive symptoms and time to recover also from secondary symptoms such as anxiety and insomnia without triggering depressive relapses.

Overall, the present study adds to accumulating evidence that depression, especially when recurrent and treatment refractory, may require a diagnostic reconceptualization based on circuit specific symptoms that can be more effectively and durably treated with targeted neuromodulation interventions.

## Authors contributions

M.F. decided on the main points of the Commentary and wrote the first draft.

H.M. and PRP provided input on the main points of the Commentary and edited the initial draft.

## Declaration of competing interest

The authors declare the following financial interests/personal relationships which may be considered as potential competing interests: Martijn Figee reports a relationship with Medtronic that includes: consulting or advisory. Martijn Figee reports a relationship with Abbott Laboratories that includes: consulting or advisory. Patricio Riva Posse reports a relationship with Abbott Laboratories that includes: consulting or advisory. Patricio Riva Posse reports a relationship with LivaNova USA, Inc. that includes: consulting or advisory. Patricio Riva Posse reports a relationship with Johnson and Johnson that includes: consulting or advisory. Patricio Riva Posse reports a relationship with Motif Neuro that includes: consulting or advisory. Helen Mayberg reports a relationship with Abbott Laboratories that includes: consulting or advisory and IP licensing. If there are other authors, they declare that they have no known competing financial interests or personal relationships that could have appeared to influence the work reported in this paper.
